# Tauopathies: Calmodulin Regulates Tau Hyperphosphorylation and Its Transformation into Disease-Specific Aggregates

**DOI:** 10.3390/biom15081133

**Published:** 2025-08-06

**Authors:** Danton H. O’Day

**Affiliations:** 1 Department of Biology, University of Toronto Mississauga, Mississauga, ON L5L 1C6, Canada; danton.oday@utoronto.ca; 2 Cell and Systems Biology, University of Toronto, Toronto, ON M5S 3G5, Canada

**Keywords:** Tau phosphorylation, CaMKII, Cdk5, GSK3B, MARK4, calcineurin, calmodulin binding domains, therapeutic approaches, Calmodulin Hypothesis, calpain

## Abstract

Tauopathies are a diverse group of neurodegenerative diseases characterized by the presence of Tau inclusions in neurons and glia. Rather than the classic steps in the transformation of Tau into neurofibrillary tangles, as first studied in Alzheimer’s disease, studies on tauopathies reveal the presence of diverse Tau aggregates that appear to be disease-specific. Regardless, the phosphorylation and hyperphosphorylation of Tau, involving various kinases and phosphatases, appear to be central to all tauopathies. As in other neurodegenerative diseases, calcium dysregulation is an early event in multiple tauopathies, where it activates calmodulin to effect downstream events. Here, the events of Tau phosphorylation and hyperphosphorylation, which involve several CaM-dependent kinases and a single CaM-regulated phosphatase, are covered. In addition, CaM has been linked to other events, including Tau aggregation. As a central player in tauopathies, CaM offers several alternative therapeutic routes that are worth investigating. For example, evidence is presented here that supports targeting specific binding motifs of key CaM-regulated Tau kinases as a novel therapeutic approach.

## 1. Introduction

Neurodegenerative diseases (NDDs) are disorders underlying cognitive, motor and/or sensory impairment that can lead to death. Tauopathy refers to a group of neurological proteinopathies that are characterized by the presence of Tau inclusions in neurons and other brain cell types [1,2]. The importance of the protein Tau in NDDs was first recognized in Alzheimer’s disease (AD), where the accumulation of intracellular tangles of Tau was linked to the disease [3,4]. A strong correlation was found between clinical symptoms and the accumulation of neurofibrillary tangles (NFTs), where NFTs are a better indicator of cognitive decline than Aβ plaques [5,6]. Based upon the locations of hyperphosphorylated Tau in the brain, the progression of AD and its associated cognitive decline correlate well with defined Braak stages for the disease [7,8]. Tau was subsequently identified as the primary biomarker and neurotoxic agent in a diversity of tauopathies [1]. In addition, Tau was detected as the secondary agent in mixed pathology forms of Huntington’s disease (HD), multiple sclerosis (MS), Parkinson’s disease (PD) and other NDDs [9,10,11].

Currently, around two dozen NDDs are recognized as tauopathies [12]. Tau-based NDDs have been subclassified as primary, with Tau accumulations as their central pathological feature or secondary when sharing other protein pathologies [13]. Primary tauopathies include aging-related Tau astrogliopathy (ARTAG), argyrophilic grain disease (AGD), globular glial tauopathy (GGT), chronic traumatic encephalopathy (CTE), primary age-related tauopathy (PART), corticobasal degeneration (CBD), Pick’s disease (PiD) and progressive supranuclear palsy (PSP) [14]. AD, PD, HD, frontotemporal dementia (FTD) with parkinsonism linked to chromosome 17 (FTDP-17) and Lewy body dementia (LBD) are secondary tauopathies. Although there can be an overlap between tauopathies, the timing, location and structure of Tau lesions in the brain vary with the specific disease [14,15,16].

Despite the central role of Tau oligomers as toxic agents in tauopathies, their formation and diverse patterns of organization and the post-translational events that drive them are still being revealed [16]. Tau protein accumulation and aggregation associated with neurodegeneration have been studied in the primary tauopathies CBD, FTDP-17, LBD, PiD and PSP [17]. PiD is characterized by the presence of phosphorylated Tau (pTau) that organizes into filamentous aggregations [18]. The pattern of Tau phosphorylation generates a different filamentous organization in Pick’s bodies, determining the degree of disease symptoms. Most information on Tau has been revealed through research on secondary tauopathies, including AD, HD and PD. pTau NFTs are present in 95–99% of individuals 80 years of age and in 70% of PD dementia patients [11,12,13,14,15,16,17,18,19]. Tau isoforms and associated Tau pathology have been found in early and late onset HD [10]. Despite this information, determining the sequence of events and the patterns of transition from one stage to another in the formation of NFTs can be confusing. An attempt here is made to simply summarize the basic sequence of events, with a focus on where protein kinases and phosphatases are known to operate. While complete sites and patterns of Tau phosphorylation related to specific tauopathies are not detailed, specific examples will be covered as they relate to topics under analysis.

While the initiating factors vary with the specific tauopathy and often remain unknown, the transition of Tau into insoluble structures follows a common path that was originally identified for AD: Tau monomers first become phosphorylated and then hyperphosphorylated as they form oligomers that organize into paired helical filaments (PHFs), which transform into NFTs [20]. The kinases and phosphatases involved in regulating Tau phosphorylation and hyperphosphorylation are discussed below. The accumulation of PHFs and NFTs in neurites and cell bodies was originally believed to cause neurodegeneration and cognitive impairment, but evidence supports Tau oligomers (TauOs) over fibrils as the primary neurotoxic agent [21]. In support of this, recent work suggests that neuron death in neurons with soluble mutant Tau occurs at three times the rate of those with NFTs [22].

It is difficult, if not impossible, to define a basic sequence of events for the progression of Tau monomers to oligomers, aggregates and fibrils for tauopathies since Tau forms diverse types of insoluble aggregates. Ultrastructural studies have shown that, in some tauopathies, Tau molecules first pair, then stack, to form long fibrils that are PHFs like those present in AD, while others are straight, as seen in CBD [12]. Others have described aggregates called “polymorphs” that appear to be tauopathy-specific [16]. In short, there is much to be learned about the diverse ways Tau can aggregate and form filaments depending on the disease. In the meantime, analyzing the role of calmodulin (CaM) as a regulator of enzymes involved in Tau phosphorylation and dephosphorylation will provide valuable information as the data from those studies is revealed. To set the stage, a basic understanding of Tau structure and the important phosphorylation and dephosphorylation targets within it is required.

## 2. Tau Structure

Understanding Tau’s localization both cytologically and in association with other cellular structures will be critical to fully understanding its role in tauopathies [23]. Tau is predominantly localized in neurons but present at lower levels in glia, astrocytes and other brain cell types [24,25]. Bound to microtubules in axons, Tau is also found unbound in the soma and dendrites of neurons [26]. Tau has long been linked to the maintenance of microtubule stability and length, but it also serves as a microtubular bundler and cross-linker, a motor protein co-ordinator, a signaling centre and a scaffolding protein [27,28]. While Tau has long been implicated in microtubule stability, its dissociation rate (40 milliseconds) means axonal Tau is constantly shifting from its microtubule-bound state to a free cytosolic state, the significance of which is under analysis [26]. Recent evidence shows that Tau is more abundant in cortical gray matter in normal and AD brains, which is dominated by soma and dendrites where soluble Tau resides, as opposed to white matter, which is rich in neuronal axons where polymerized tubulin microtubules predominate [29].

Multiple recent reviews have discussed the genetics of Tau as well as the generation of Tau types based upon alternative splicing and post-translational modifications [12,30,31]. The microtubule-associated protein Tau (*MAPT*) gene on chromosome 17 codes for Tau protein [12]. The revelation that mutations in the *MAPT* gene that encodes Tau can cause neuronal dysfunction, cognitive impairment and death in frontotemporal dementia (FTD) and PSP sufferers supported the importance of tauopathy as a significant class of NDDs [30,32]. The generated Tau mRNA (*MAPT* mRNA) undergoes alternative splicing, resulting in six isoforms (0N3R, 1N3R, 2N3R, 0N4R, 1N4R, and 2N4R) that are differentially expressed in neurons of the adult brain [33]. The longest isoform, 2N4R, is particularly significant in AD and other tauopathies because of its role in microtubule stabilization and its potential to aggregate.

**Figure 1:**
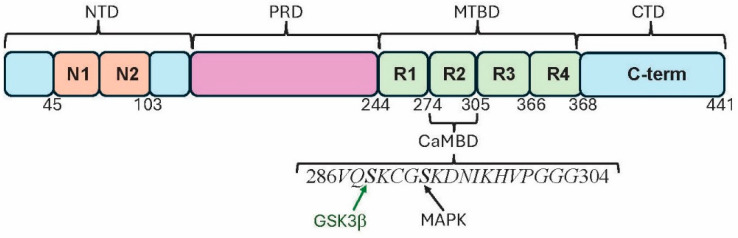
The basic organization of 2N4R, the longest Tau isoform. The four well-studied, primary regions N-terminal domain (NTD), proline-rich domain (PRD), microtubule-binding domain (MTBD), and C-terminal domain (CTD) are all shown. In addition, the experimentally identified CaM-binding domain (CaMBD) is indicated. Serine residues in the CaMBD are phosphorylated by the key phosphatases glycogen synthase kinase 3 beta (GSK3β) and mitogen-activated protein kinase (MAPK).

The microtubule binding of Tau is mediated by the R1-4 domains in the microtubule-binding domain (MTBD), while the proline-rich domain (PRD) is linked to microtubule stabilization and interactions with other cellular constituents (Figure 1) [34]. Splicing can exclude microtubule-binding regions in the MTBD, resulting in different isoforms [35]. N refers to the two N-term isoform sequences that are possible as either none (0N), one (1N) or two of them (2N) being present. The R2 and R3 regions of Tau are not only linked to microtubule binding but can also seed Tau aggregation and drive its propagation [36]. 3R Tau isoforms are missing the second MTBD, while 4R Tau isoforms contain all four MTBDs. Brain levels of 3R and 4R isoforms are essentially equal, with different tauopathies exhibiting filaments with different isoforms of Tau, causing different folding patterns. For example, AD has 4R Tau in its filaments, while PiD has 3R Tau [37].

## 3. Calcium Dysregulation in Tauopathies

Calcium dysregulation is a well-studied, early, common and critical event in most, if not all, NDDs, including secondary tauopathies (e.g., AD, HD, PD) [38,39,40,41]. Calcium dysregulation underlies Tau pathology [42]. In AD, the increased calcium levels induce pTau formation and aggregation [43]. Two primary calcium targets relevant here are calpains and CaM. Calpains are involved in NDDs, where they alter critical proteins, cleave and activate CaM-dependent phosphatase calcineurin (CN; PP2B) CN, protein kinase Cα and glycogen synthase kinase 3β, and affect αSyn aggregation in PD [44]. Calpain also regulates Tau phosphorylation by cleaving and activating cyclin-dependent kinase 5 (Cdk5), as detailed below.

In contrast, CaM has a diverse number of functions in primary and secondary tauopathies. In neurons, the cytosolic level of calcium ions (1.1–1.4 mM) is regulated by receptors (e.g., metabotropic glutamate receptor (mGluR)) and ion channels (e.g., N-methyl-D-aspartate receptor (NMDAR), plasma-membrane Ca^2+^-ATPase (PMCA), store-operated calcium channels (SOCCs)) in the plasma membrane and in the endoplasmic reticulum (e.g., ryanodine receptor (RyR)), lysosomes (e.g., TRP ion channel family) and mitochondria (e.g., Na^+^/Ca^2+^ exchanger (NCX); voltage-dependent anion channel (VDAC)) [38,45,46,47]. At least three of these regulators of cytosolic calcium levels bind to and are regulated by both CaM and Tau (PMCA, RyR, VDAC) [41]. Thus, CaM can feed back to affect calcium levels and, as a result, its own function in NDDs. Moreover, pathological Tau’s complex role in calcium dysregulation is demonstrated by its disruption of septin filament organization, which activates store-operated calcium channels (SOCCs), causing further increases in intraneuronal calcium levels [48]. Pharmaceuticals that stabilize septin filaments prevent toxic calcium entry through SOCCs and restore long-term potentiation in cellular models of Tau pathology [49]. Adding to this complexity, SOCC function is also regulated by Ca^2+^/calmodulin-dependent inactivation (CDI), as previously reviewed [50].

Comparatively, the information on calcium in primary tauopathies is sparse, with recent major reviews failing to mention the importance of the cation at all [51,52]. That said, the few studies of primary tauopathies that have been conducted support concepts developed for secondary tauopathies and other NDDs that led first to the Calcium Hypothesis and subsequently to the Calmodulin Hypothesis [53,54,55]. These primary tauopathies include ARTAG, CTE and PART. A phospho-proteomic analysis conducted for aging-related Tau ARTAG revealed differences in differential phosphorylation between neuronal and glial proteins [56]. Of relevance here was that proteins involved in calcium/calmodulin signaling were identified as being upregulated, including calcium/calmodulin-dependent 3′,5′-cyclic nucleotide phosphodiesterase 1C, calcium/calmodulin-dependent protein kinase type II subunit gamma and voltage-dependent P/Q-type calcium channel subunit alpha-1A. Thus, calcium and calmodulin are intimately associated with ARTAG, yet continued research has failed to advance our understanding of this signaling. CTE, also referred to as traumatic brain injury (TBI), is a progressive neurodegenerative disease caused by repetitive head trauma [57]. The impact of brain damage resulting in calcium dysregulation, the ion channels involved and the downstream function of calcium–calmodulin signaling have recently been detailed [50]. Although PART does not form amyloid plaques, calcium dysregulation is an early event associated with Tau pathology, and this tauopathy shares transcriptional changes in CA1 pyramidal neurons that are similar to those observed in AD [58].

Tau is known to feed back to increase calcium dysregulation in primary tauopathies. For example, TauO causes increases in intracellular calcium levels and neuron death in FTLD-Tau [59]. At least two ion channels bind to Tau: plasma-membrane Ca^2+^-ATPase (PMCA) and voltage-dependent anion channel 1 (VDAC1). CaM binds to and activates plasma-membrane PMCA, while Tau binds and inhibits it [41,60]. Mitochondrial VDAC1 pore function is blocked by Tau binding, while CaM binding reduces conductivity by affecting gating and permeability [61,62]. Since Tau also binds to CaM, as discussed below, the potential for complex feedback mechanisms involving PMCA and VDAC in regulating calcium dyshomeostasis in tauopathies exists and, likely, will prove to be complex.

## 4. Calmodulin: The Basics

A small (148 amino acid aa, 16.7 kDa), highly conserved protein, CaM is expressed at high levels in neurons, where it binds to two distinct CaM-binding protein (CaMBP) populations: calcium-independent and calcium-dependent CaMBPs. Target binding to CaM does not involve conserved binding sequences but is primarily based on patterns of hydrophobic amino acids [63,64]. Calcium-free CaM (ApoCaM) ApoCaM binding involves the IQ motif ([FILV]Qxxx[RK]Gxxx[RK]xx[FILVWY]) and IQ motif variants that oversee calcium-independent binding. Calcium binding causes a dramatic transition in a condensed apoCaM shape to a more extended, barbell-like Ca^2+^/CaM shape. This opens hydrophobic binding regions that can bind to variable canonical sequences of hydrophobic amino acids in an ~18–22 sequence primarily defined by anchor hydrophobic amino acids in specific locations (e.g., 1-10, 1-5-10, 1-12, 1-8-14, 1-16). That said, many non-canonical binding domains have also been discovered [65]. Algorithmic predictions can effectively identify calcium-dependent, canonical CaM-binding domains (CaMBDs) with high probability, but the binding domains must be validated through experimental analysis [66].

## 5. Tau Binds to Calmodulin

Tau binds to Ca^2+^/CaM in vitro, an event that prevents Tau from binding to microtubules, most likely because the CaMBD is located within the microtubule-binding sequence (Figure 1) [67,68]. Ca^2+^/CaM binds to the second tubulin binding site repeated in Tau, and CaM binding to Tau also inhibits its phosphorylation by protein kinase C. Thus, the previously identified inhibition of tubulin polymerization into microtubules by Ca^2+^/CaM may be due in part to these events. Since the precise CaM-binding domain (CaMBD) in Tau had not been previously detailed, a Calmodulin Target Database scan of the R2 sequence was carried out here. This database is recognized as the “gold standard” for determining putative CaM-binding domains (CaMBDs) [69,70]. For example, Calmodulin Target Database scanning of proteins involved in AD identified presumptive CaMBDs in amyloid precursor protein 1 (APP1), β-secretase 1 (BACE1) and presenilin-1 (PSEN1), the source of Aβ and two key enzymes in amyloidogenesis, respectively [54]. All were subsequently experimentally validated as true CaMBPs [71,72,73]. The scan of the full Tau sequence carried out here revealed a 1-14 binding motif (286VQSKCGSKDNIKHVPGGG304; 1-14 hydrophobic amino acids are underlined) in the R2 domain. It remains to be determined if CaM binding to Tau is affected by the phosphorylation of any of the three phosphorylation sites, of which two (Ser289 and Ser293) are phosphorylated in AD [74]. This information will be useful for further analyses of the role of CaM in Tau function in various tauopathies.

## 6. Tau Phosphorylation

In healthy brains, Tau is primarily soluble and unfolded, but it becomes post-translationally modified to form aggregates in NDDs [75]. In addition to phosphorylation, many other post-translational modifications are open to Tau: acetylation, ubiquitination, glycation, glycosylation, SUMOylation, methylation, oxidation and nitration [31,76]. Each of these post-translational modifications (PTMs) has been linked to tauopathies, including AD and FTLD-Tau [31]. With an eye to the development of new therapeutic strategies, Hong and colleagues have reviewed the post-translational modifications (PTMs) acetylation, glycosylation, hyperphosphorylation, lysine methylation and ubiquitination in AD and their roles in neuroinflammation, microtubule structure, mitochondrial malfunction and synaptic dysfunction [77]. For example, both acetylation and ubiquitination of Tau occur in AD. Acetylation functions in Tau turnover and insolubility, while ubiquitination is involved in Tau clearance [12]. In AD, Tau acetylation impairs microtubule-binding and drives Tau aggregation, specifically with acetylated K280 Tau locating within aggregates [77]. In addition, PHFs from the AD brain are highly ubiquitinated—an event that may be involved in NFT formation [78]. However, the significance of CaM as a regulator of Tau acetylation, ubiquitination and other PTMs in tauopathies remains to be studied. That said, most research has focused on Tau phosphorylation due to its validated importance in AD and multiple tauopathies.

Hyperphosphorylation of Tau is sufficient to drive the formation of Tau filaments [79]. In addition, there is also a relationship between specific tauopathies, Tau phosphorylation and filament structure [80]. Research has used electron cryo-microscopy to reveal disease-specific structures of tau filaments in tauopathies, including AD, CTE, CBD, GGT, PiD and PSP, although some overlap was seen for GGT and PSP [80]. This information, coupled with specific details about the types of folding and the phosphorylation events that are involved, may lead to a better understanding of the pathogenesis of tauopathies. How does Tau become hyperphosphorylated, and what is the role of CaM in this event? It has been suggested that Tau is one of the most difficult phosphoproteins to study [81]. This is because, with 45 Ser, 35 Thr and 5 Tyr target residues for Tau kinases, it has 85 potential phosphorylation targets in its longest isoform (2N4R; 441aas) [79]. Thus, the potential combination of sites that could be involved in tauopathies is “astronomical” [81]. As revealed for AD, glycogen synthase kinase 3 beta (GSK3β), Cdk5 and p38 MAPK are the major Ser/Thr kinases involved in Tau phosphorylation, with DYRK1A and microtubule affinity-regulating kinase 4 (MARK4) having only a few targets, as summarized in the following list: GSK3β (43 Ser, Thr sites), Cdk5 (13 Ser, Thr), p38 MAPK (19 Ser, Thr), DYRK1A (3 Ser, Thr) and MARK4 (2 Ser, Thr) [79,82,83]. In contrast, Tyr is phosphorylated at a limited number of sites by Src (1 Tyr site), Fyn (1 Tyr), c-Abl (1 Tyr) and Rho-associated protein kinase (ROCK; 3 Tyr). Of course, identifying those phosphorylated amino acids that are critical, as has been done for AD (e.g., Ser202, Thr205, Tyr18, Ser199, Thr231 and Ser422), is key since it points to specific therapeutic targets [35]. Therapeutic interest is currently focusing on GSK3β, Cdk5, MAPK, MARK4, ROCK, Ca^2+^/CaM-dependent protein kinase II (CaMKII) and others that are involved in Tau hyperphosphorylation, especially in AD [84]. The level of Tau phosphorylation also depends on dephosphorylation. In AD, dephosphorylation by multiple protein phosphatases (PP-1, PP5, PP2A, PP2B and PTEN (phosphatase and tensin homolog deleted on chromosome 10)) has been observed [85].

Here, the interest is on the role of CaM in Tau phosphorylation, an event that is dictated by the contrasting activities of several CaM-regulated kinases and one CaM-regulated phosphatase: Four CaM-binding kinases, CaMKII, Cdk5, GSK3β and MARK4, and CN Ca^2+^/CaM-dependent calcineurin (CN; PP2B) have been shown to phosphorylate and dephosphorylate Tau, respectively [76,86]. ROCKs regulation by CaM remains to be clarified. Each of these enzymes and their proven or putative regulation by CaM in Tau phosphorylation is examined here.

**Figure 2:**
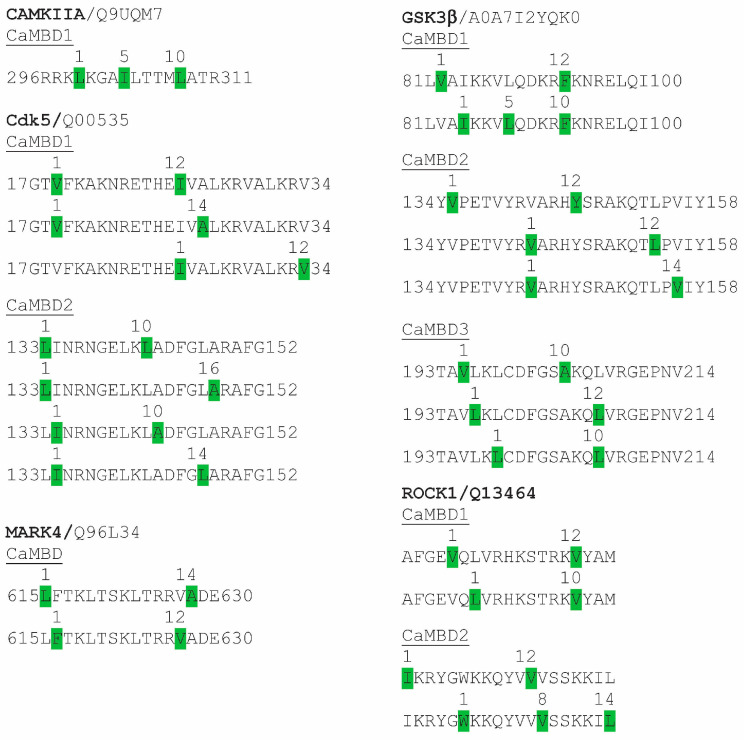
Calmodulin binding domains and binding motifs in tauopathy kinases. The kinase name and Uniprot ID are indicated (e.g., CaMKIIA/Q9QUM7), with the identified CaM-binding domains (CaMBD1-3) listed below them. The numbers at the start and end of each CaMBD indicate the position of the domain in the protein sequence. Numbers above the green-highlighted hydrophobic amino acids in the CaMBDs indicate specific binding motifs. Individual CaMBDs can have multiple binding motifs within them. Abbreviations. CaMKIIA, Ca^2+^/CaM-dependent protein kinase alpha; Cdk5, cyclin-dependent kinase 5; GSK3b, glycogen synthase kinase 3 beta; MARK4, microtubule affinity-regulating kinase 4; Rho-associated protein kinase 1 (ROCK1).

In addition to summarizing past research on kinases that have shown proven or potential regulation by CaM, CaMKIIA, Cdk5, GSK3β, MARK4 and ROCK1 have been scanned here using the Calmodulin Target Database to determine the location and attributes of potential CaM-binding sites. Subsequently, identified CaMBDs were visually scanned to identify specific binding motifs, as noted in Figure 2. These results support and extend earlier analyses of the CaMBDs of Cdk5 and CaMKII [55].

### 6.1. CaMKII

Ca^2+^/CaM binds to and activates CaMKII [87]. CaMKIIA possesses a single CaMBD (296RRKLKGAILTTMLATR311) [88]. Here, the identical CaMBD was detected using the Calmodulin Target Database, within which a single 1-5-10 binding motif was visually detected (Figure 2). In addition to its multiple functions in AD and other NDDs, CaMKII has been shown to localize to CA1 neurons and to phosphorylate Tau at specific sites: Thr212, Ser214, Ser262, Ser356, Ser131, Thr135, Thr212 and Ser214 [89,90,91,92]. Of these, Ser262 and Ser256 are critical amino acids involved in the initiation of Tau aggregation [93,94]. Immunostaining for S262 and S356 localizes to prefibrillar/granular Tau aggregates in hippocampal pyramidal neurons and thus can serve as an early biomarker in NFT formation in AD [94]. As detailed below, CaMKII is involved in prephosphorylation events, setting the stage for subsequent hyperphosphorylation by other kinases, including Cdk5 and GSK3β [95]. As covered in the Discussion section, numerous inhibitors and activators of CaMKII have therapeutic value [92].

### 6.2. GSK3

Early studies on GSK from various sources revealed it as a CaMBP. Forty-five years ago, a rabbit liver cAMP-independent GSK was shown to require Ca^2+^/CaM for its activity [96]. As a CaM-dependent kinase, it specifically phosphorylated glycogen synthase, partially inactivating it, an event inhibited by calcium chelation and by the CaM antagonist trifluoperazine. Three years later, a Ca^2+^/CaM-regulated GSK from skeletal muscle was identified [97,98]. That enzyme has been recognized as a member of the GSK3 family [99]. Despite the early recognition of CaM regulation of GSK3 enzyme activity, the importance of CaM’s role in GSK regulation has been ignored. To further assess the potential of GSK3β as a CaMBP, a Calmodulin Target Database scan of the protein was carried out to determine if it contained presumptive CaMBDs. The scan revealed that GSK3β contains three well-separated CaMBDs, each with multiple binding motifs (Figure 2). CaMBD1 (81LVAIKKVLQDKRFKNRELQI100) has two motifs (1-12; 1-5-10), while CaMBD2 (134YVPETVYRVARHYSRAKQTLPVIY158) has four motifs (two 1-12; one 1-14). CaMBD3 (193TAVLKLCDFGSAKQLVRGEPNV214) has three motifs (two 1-10; one 1-12). Thus, there is strong evidence that GSK3β binds to and is regulated by CaM. That said, CaM also indirectly regulates GSK3 via CaMKII, which phosphorylates GSK3, causing its inhibition [100].

### 6.3. Cdk5

Cyclin-dependent kinase 5 (Cdk5) is a proline-directed serine/threonine kinase shown to be involved in Tau hyperphosphorylation, aggregation and NFT formation in AD [101,102]. In neurons, Cdk5 regulates numerous critical functions, making it stand alone as a diversified neuronal kinase [103]. Cdk5 phosphorylates Tau at T181, S202, T205, T212, T217, S235, S369 and S404, four of which (pSer202, Thr205, Thr231, pSer396) are hyperphosphorylated in post-mortem AD brain samples [104,105,106]. Cdk5 silencing using RNA interference resulted in reduced levels of both Tau phosphorylation and NFT formation in a transgenic mouse model [107]. Cdk5 works in association with p35 and p39 as a Cdk5/p35 complex [108]. In NDDs, increased calcium activates the calcium-dependent protease calpain, which cleaves p35 to p25, forming a more stable and more active Cdk5/p25 complex that hyperphosphorylates Tau [108,109].

Cdk5 was first identified as a CaMBP in the eukaryotic microbe *Dictyostelium discoideum* [110]. Deletion of one of the two CaMBDs in *Dictyostelium* Cdk5 (132LLINRKGELKLADFGLARAFGIP154) prevented CaM binding, validating it as a CaMBP. Furthermore, the Calmodulin Target Database scanning conducted here revealed two highly conserved CaMBDs in human Cdk5, each with multiple CaM-binding motifs: CaMBD1 (17GTVFKAKNRETHEIVALKRVALKRV34) has two motifs (two 1-12; one 1-14), while CaMBD2 (133LINRNGELKLADFGLARAFG152) contains four (two 1-10; one 1-16; one 1-14) (Figure 2).

### 6.4. MARK4

Primarily expressed in brain, where it phosphorylates Tau, MARK4 is an AMP-activated protein kinase (AMPK) belonging to the subfamily of Ca^2+^/CaM-dependent protein kinases (CaMKs) [79]. There are four MARKs, of which MARK4 has been the focus of research on Tau phosphorylation [84]. A mutated form of MARK4 has been linked to the occurrence of early onset AD [111]. Research has shown that MARK4 can work with Cdk5 in an “activation loop”, significantly increasing Tau phosphorylation at Cdk5 and MARK targets, including Ser262 [112]. MARK4 activity is increased in AD brains, where it hyperphosphorylates Ser262, after which GSK3β, Cdk5, MAPK and other kinases can increase the level of hyperphosphorylation [84,113]. Saito’s work also revealed that Cdk5 activity is required for a feedback loop involving the phosphorylation and activation of MARK4 [112]. Analyses using the Calmodulin Target Database carried out here support earlier work indicating that MARK4 is a CaMBP, since a single CaMBD was detected that contains two binding motifs (one 1-14; one 1-1-12) (Figure 2) [84]. Despite the fact that MARKs are CaM-dependent kinases that are potential therapeutic targets in AD, there is no evidence that CaM regulation of MARK4 is a current therapeutic target.

### 6.5. ROCK and Rho

The involvement of CaM in the regulation of Rho-associated protein kinase (ROCK) is being evaluated [114]. ROCK and ras homolog gene family member A (RhoA), which is activated by ROCK, work together mediating numerous cellular processes, but abnormal activation underlies various diseases [115]. In fibroblasts, increased levels of cytosolic calcium activate RhoA/ROCK [114]. The inhibition of this activation by treatments with a CaM antagonist (W7) or CaMKII inhibitor (KN506) suggests that CaM/CaMKII signalling regulates RhoA/ROCK function [114]. By removing CaMKII autoinhibition, Ca^2+^/CaM binding activates the Ser/Thr kinase activity of the enzyme [116]. A question remains: is CaM acting directly on ROCK, or is its effect via its activation of CaMKII? Preliminary analyses suggest the former is possible, since a Calmodulin Target Database analysis revealed that ROCK1 has two potential CaMBDs situated at opposite ends of the protein. A visual scan of CaMBD1 (86AFGEVQLVRHKSTRKVYAM105) indicated two binding motifs (1-12; 1-10), while CaMBD2 (1133IKRYGWKKQYVVVSSKKIL1151) also had two (1-12; 1-8-13).

### 6.6. Calcineurin

The phosphorylation of Tau by kinases is counterbalanced by its dephosphorylation by protein phosphatases. Calcineurin (CN), also known as protein phosphatase 2B (PP2B) and protein phosphatase 3 (PPP3), is the sole Ca^2+^/CaM-dependent serine/threonine protein phosphatase [117,118]. Expressed at high levels in neurons, CN is involved in apoptosis, learning, memory and synaptic plasticity. CN is widely expressed in the brain, with enhanced localization in postsynaptic dendritic spines [119]. After early studies revealed a role for CN in Tau dephosphorylation, continued research suggests it might only have minor importance since, compared to other phosphatases, it is only involved in about 7% of Tau dephosphorylation events [120]. Of course, quantity is not as important as quality, so understanding the specific contribution of CN dephosphorylation is essential.

CN from AD brains differentially dephosphorylates pTau at very high levels for Ser262 in the R1 domain (63% decrease in phosphorylation) and Ser396 in the R4 domain (78%) but less effectively for S199 (38%), T217 (32%) and S422 (32%) [121]. Thus, CN selectively dephosphorylates Ser-262 and Ser-396 of pTau.

### 6.7. Bottom of Form

The potential significance of CN in Tau phosphorylation correlates with its increased activity in AD brains reported by different research groups [120,122]. Early immunological studies showed no detectable decrease in concentration or alterations in CN localization in AD brains, but CN activity was shown to decrease slightly in one study [123]. That detected decrease was likely due to the non-specific substrate (p-nitrophenyl phosphate) and assay method used, since assays using P32-labelled Tau support an increase in CN activity in AD brains [120]. Part of this increase is due to calpain I, which increases in activity in response to the unregulated calcium levels that characterize AD and other NDDs [50,120]. CN is a heterodimer consisting of A and B subunits, of which A binds to CaM to activate the phosphatase activity [117,124]. Calpain cleaves the A subunit, making it CaM independent, enhancing its phosphatase activity and making it irreversibly hyperactive [120,122]. The previously identified region of CaM binding (391ARKEVIRNKIRAIGKMARVFSVLR414) was verified here using the Calmodulin Target Database and a visual scan that identified five different binding motifs (1-16; 1-8-14; 1-14; 1-12; 1-10) within the sequence.

Both CNA and CNB, the subunits of CN, bind to Tau [86]. The CNB subunit of CN binds the MTBD of Tau, while the PRD mediates the binding of the full CN molecule to Tau [125]. However, other factors appear to play a role in the regulation of CN activity and Tau dephosphorylation, which might explain any reported differences, since there is a complex interplay between the CN subunits CNA and CNB, CaM, Tau and CN enzyme activity, as discussed below [86]. While CaM was shown to inhibit the interaction between CN and Tau, it also enhances CN activity several-fold. As these regulatory element interactions are resolved for AD and other tauopathies, the dephosphorylation of Tau by CN presents a therapeutic route that is worth further consideration.

## 7. From Prephosphorylation to Hyperphosphorylation

**Figure 3:**
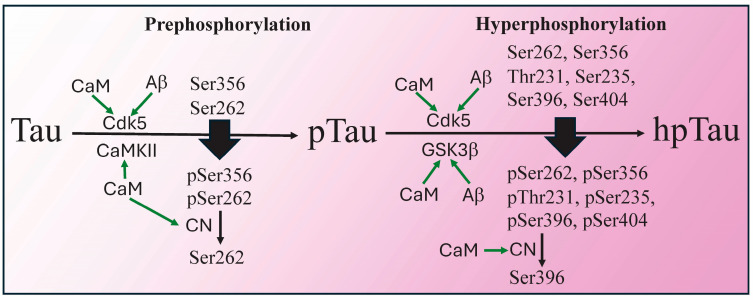
CaM-regulated kinases and CN are involved in the prephosphorylation and hyperphosphorylation and dephosphorylation of Tau, as detailed in the main body of the review. In a prephosphorylation step, the Ca^2+^/CaM kinase II (CaMKII) and cyclin-dependent kinase-5 (CDK-5), which is regulated by both CaM and amyloid beta (Aβ), phosphorylate the amino acids Ser356 and Ser262 in Tau. CaM also activates calcineurin (CN) that can dephosphorylate Ser260. Prephosphorylation opens amino acids to phosphorylation by Cdk5 and glycogen synthase kinase-3β (GSK3β). GSK3β is activated by Aβ and, as detailed in the text, there is strong evidence that it binds to and is regulated by CaM. CN, when activated by CaM, is able to dephosphorylate Ser396.

Tau hyperphosphorylation begins with prephosphorylation events that facilitate subsequent hyperphosphorylation (Figure 3). For example, Ser262 and Ser356 are prephosphorylated by Cdk5 and CaMKII, both of which bind to and are regulated by CaM [110,126]. Phosphorylation of these two amino acids is believed to alter the conformation of Tau such that Ser262 and Ser356, along with multiple other disease-linked phosphorylation sites (e.g., Thr231, Ser235, Ser396, Ser404), become open to further phosphorylation by the CaMBP GSK3β and other kinases [126,127]. Amino acids associated with both prephosphorylation (Ser262) and hyperphosphorylation (Ser396) are targets for dephosphorylation by CaM-regulated CN. The prephosphorylation of Ser262 by the CaMBP MARK4 as a prelude to its hyperphosphorylation is covered above. While Tau also binds to CN, as described above, this binding is prevented by CaM, an event not indicated on Figure 3 since its significance remains to be discovered.

## 8. Tau Aggregation

pTau monomers are soluble and non-toxic, but aggregation causes them to become toxic. Hyperphosphorylation is sufficient to drive Tau aggregation into paired helical filaments and straight filaments, but other factors also come into play [17,27,128]. Tau monomers demonstrate a “paperclip” conformation in which the N-term and C-term are folded, causing them to reside close to the MTBD, a shape that inhibits Tau aggregation [17]. Since displacing the C-term/MTBD interaction can initiate aggregation, does CaM binding to the MBTB disrupt C-term binding and facilitate aggregation? This question remains to be answered. On the other hand, Aβ is a CaMBP that accelerates both pTau hyperphosphorylation and its oligomerization/aggregation. It does so initially by activating two central Tau kinases, Cdk5 and GSK3β, as summarized in Figure 3 [129,130]. Thus, CaM is involved in different ways in Tau hyperphosphorylation, oligomerization and aggregation (e.g., via kinase regulation and Aβ actions), thus opening up previously unstudied therapeutic routes. pTau aggregation also involves enzyme-directed aggregation involving CaM-regulated transglutaminase.

Tissue transglutaminase functions as a transamidase, GTPase, protein disulfide isomerase and protein kinase [131]. As a transamidase, it generates cross-linking between the γ-carboxamide of glutamine and the ε-amino group of lysine, transforming soluble proteins into insoluble, protease-resistant complexes [131,132]. TGM2 stands out because it is involved in the aggregation of all the major toxic biomarker proteins for AD, HD, PD and LBD (Aβ, αSyn, mHTT, Tau) [132,133]. Elevated levels of TGM2 have been shown in pathogenic stages of AD and other NDDs where the enzyme co-localizes with Tau aggregates, PHFs and NFTs [134,135,136]. Multiple studies have verified the role of TGM2 in the aggregation of Tau [134,137,138,139]. TGM cross-links pTau into oligomers and PHFs via a few discrete sites [137]. In vitro, TGM2 mainly targets Gly and Lys residues within or adjacent to the MTBD [132]. Support for the TGM2 cross-linking of Tau was garnered from experiments on brain tissue from PSP and AD patients, which revealed “significantly higher levels of ε–(γ-glutamyl) lysine cross-linking of PHF-tau” in disease-specific brain regions [138].

Transglutaminase is a CaMBP, and Ca^2+^-CaM binding increases TGM2 activity up to 3-fold [140,141]. Two CaMBDs were previously identified in human TGM2 [142]. CaMBD1 (414KSINRSLIVGLKISTKSVGR433) possesses three binding motifs (1-16, 1-12, 1-5-10), while CaMBD2 (665VVNFESDKLKAVKGFRNVII683) has five motifs (two 1-12, 1-8-14, two 1-10). Traditional approaches to developing a TGM2-based therapy (e.g., antibodies, siRNA, active site inhibition) have been unsuccessful. This suggests that a greater understanding of the role of CaM regulation of TGM2 and Tau transformation might open new therapeutic opportunities.

## 9. Discussion

Calcium dysregulation is a well-documented phenomenon that is common to most (if not all) NDDs [40,41,53]. CaM is a primary neuronal downstream target of calcium ions, which led to the Calmodulin Hypothesis of AD, where CaM was shown to be involved in multiple aspects of amyloidogenesis and other critical events of the disease [54]. The Calmodulin Hypothesis of AD was subsequently extended to include HD, PD, MS, TBI and other NDDs, making it a potentially universal hypothesis [142]. That idea is supported here with evidence that CaM has a central role in tauopathies. Recent work continues to support CaM-regulated enzymes in Tau phosphorylation. For example, a diet rich in saturated fatty acids (e.g., palmitic acid) causes increased brain aging and multiple post-translational modifications of Tau associated with AD [143]. A review of the literature also revealed that palmitic acid-dependent metabolic stress induces kinases and phosphatases involved in Tau phosphorylation, including proven CaM-regulated kinases (i.e., CaMKII, GSK3β, MARK2) and the CaM-regulated phosphatase CN, as discussed above [144].

This review extends these observations by showing that CaM is not only involved in regulating key enzymes involved in Tau phosphorylation but also other critical events in tauopathies. With a CaMBD in Tau’s R2 domain in the MTBD, CaM mediates microtubule binding, potentially affecting microtubule structure as well as Tau seeding and propagation. CaM impacts Tau phosphorylation, hyperphosphorylation and aggregation in tauopathies via a number of different routes. CaM directly binds to and regulates key enzymes in Tau phosphorylation and hyperphosphorylation. Of these CaMKII, Cdk5, MARK4 and GSK3β are proven CaMBPs. CaM directly and indirectly regulates both prephosphorylation (e.g., via Cdk5 and CaMKII) and hyperphosphorylation events (e.g., via Cdk5, GSK3β). CaM also functions in Tau dephosphorylation, since it binds to and activates CN. These multiple direct and indirect functions of CaM in Tau phosphorylation and aggregation thus offer unique therapeutic routes for tauopathies.

Of the limited number of clinical trials targeting Tau, none have provided sufficient therapeutic value to be useful [13]. However, current research continues to suggest various treatment options. Dauricine and daurisoline, two bisbenzylisoquinoline alkaloids in the dried rhizome of *Menispermum dauricum* DC used in Chinese medicine, are strong autophagy blockers [145]. Treatment of Glu-injured and Aβ_25-35_-injured PC12 cells with these natural alkaloids decreased calcium levels and subdued the Ca^2+^/CaM /CaMKII pathway, inhibiting Tau phosphorylation. FK506 is an FDA-approved CN inhibitor that has been effectively and safely used for two decades to prevent organ rejection, so repurposing it for research into tauopathies still stands out as a therapeutic option [146]. Experiments in TauO-treated mice revealed that FK506 inhibition of CN reduced hippocampal Tau levels and synaptic damage [8]. A previous study had also shown a reduction in AD pathology in mice treated with FK506 [147]. Independent studies in humans and mice have shown that FK506 prevents the “toxic effect of brain-derived TauO on synaptic plasticity” [146,148]. Inhibition of CN with antisense oligonucleotides and enzyme-specific pharmaceuticals (e.g., FK506) leads to Tau hyperphosphorylation involving residues Ser262, Ser369, Thr181 and Thr231 [149,150]. Similarly, AβO and αSynO increase the hippocampal activity of CN, memory loss and synaptic remodeling, events that are restored by treatments with FK506 [151,152,153]. pTauO, which induces neurodegeneration, also enhances hippocampal CN activity, while treatment with FK506 inhibits this increase [8]. Understanding the effects of calcineurin inhibition in other tauopathies should be investigated as well.

CaMKII is involved in innumerable cellular processes, of which its central role in learning, memory and diminishment in AD and other tauopathies is of central interest [154]. CaM-based inhibition of CaMKII using KN62, KN93 and other isoquinolinesulfonamide derivatives has long been employed in the treatment of various diseases and in the dissection of CaMKII’s role in neuron function [154,155]. However, while having provided useful insight, the KN derivatives also inhibit CaMKIV, VDCCs, voltage-dependent K channels and other targets, making their use imprecise at best. The pharmacological inhibition of CaMKII and CN is not without side effects [156]. Despite some success with less-than-selective inhibitors, it is possible that targeting CaMKII and CN, as well as other key Tau kinases, could benefit from developing peptides based on the binding motifs in their CaMBDs. It seems reasonable that precise targeting of key kinases and CN could lead to more effective treatment with fewer side effects.

**Figure 4:**
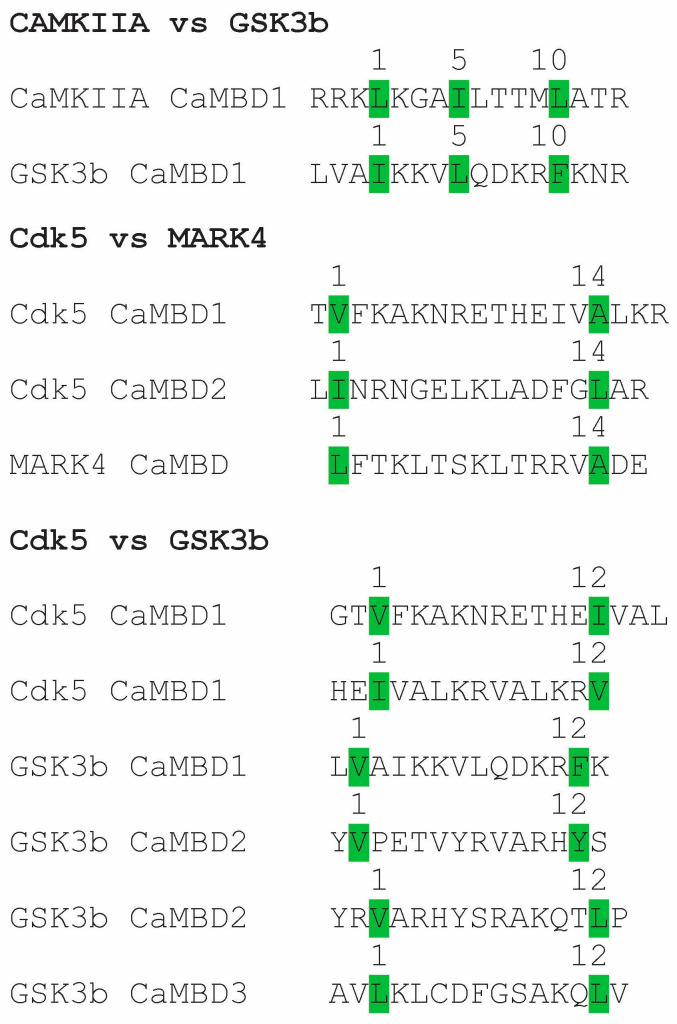
Calmodulin binding motifs in Tau kinases. The numbers above the amino acid sequences indicate binding motifs; key hydrophobic amino acids are highlighted in green. CaMKIIA, Ca^2+^/CaM-dependent protein kinase 2A; Cdk5, cyclin-dependent kinase 5; GSK3b, glycogen synthase kinase beta; MARK4, microtubule affinity-regulating kinase 4.

As discussed above, CaM is recognized for its lack of traditionally defined binding motifs for calcium-mediated protein interactions, but that view may be more apparent than real. Overall, CaM-binding domains are currently defined by sequences involving anchor hydrophobic amino acids (Phe, Ile, Leu, Val and Trp) at the start and end of the functional binding motif, leading to a select number of basic patterns. By 2000, 300 CaMBPs had been identified, and their binding domains are revealed as listed on the Calmodulin Target Database [69]. However, the essential hydrophobic amino acids in each binding motif in those CaMBDs remain to be studied for most of those proteins. Analysis of the binding motifs within specific CaMBPs could reveal unique, enzyme-specific patterns. Here, a series of potential binding motifs has been identified from four key Tau kinases (Figure 4). Precise targeting of these kinases by focusing on CaM-binding domains has not been previously proposed, but a few comparisons suggest this novel route to developing Tau kinase-specific inhibitors could be a route worth exploring.

The comparison of the key hydrophobic residues in three binding motifs of pairs of four Tau kinases (CaMKII, GSK3β, Cdk5, MARK4) from Figure 3 reveals no overlap in key residues (Figure 2). Thus, the key 1-5-10 residues for CaMKII (LIL) differ from those in the 1-5-10 motif of GSK3β (ILF). Two 1-14 motifs of Cdk5 (VA, IL) were different from the 1-14 motif of MARK4 (LA). Cdk5 possesses two 1-12 motifs (VI, IV), which differ from the four 1-12 motifs found in GSK3β (VF, VY, VL, LL). These preliminary analyses indicate that key CaM-binding Tau kinases have unique arrangements of anchor hydrophobic amino acids that serve to delineate them and, as a result, could be used to develop kinase-specific drugs to target them. Using machine learning and artificial intelligence to more critically analyze and compare binding motif sequences from all CaM-binding Tau kinases could generate target sequences for the development of precise targeting in tauopathies, as has been accomplished for biomarker analysis in AD [157].

There is still much basic work to be undertaken to improve our understanding of CaM’s roles in tauopathies and other NDDs. Otherwise, our understanding of events critical to the onset and progression of tauopathies will remain incomplete. Determining CaM levels, locations and co-locations with CaM-binding biomarkers in the brain is essential. What are the key amino acids involved in Tau binding to CaM, and can they be used to develop antagonists to fully understand the importance of this interaction? How important is CaM regulation of key enzymes in the phosphorylation state of specific amino acids? Are key amino acids whose phosphorylation depends on CaM enzymes, such as Ser262 and Ser356, critical to multiple tauopathies, or are certain sites disease-specific, in turn driving the formation of tauopathy-specific polymorphs? Is the CaM-regulated, TGM2-mediated aggregation of pTau a common and critical event in all, or just specific, tauopathies?

## 10. Conclusions

It has been acknowledged that “Among significant obstacles to the development of effective therapeutic strategies is the lack of a refined understanding of the signaling pathways that are relevant to diverse biological and pathological processes in each of the tauopathies, as well as their regulation” [158]. We have made the case here that CaM and its CaMBP, key components in calcium signaling, conduct critical functions in tauopathies, making them prime candidates for further investigation into their functions and use as therapeutic targets. We have made the case that CaM regulates key enzymes involved in the phosphorylation (i.e., kinases CaMKII, GSK3β, Cdk5, MARK4 and the phosphatase CN) and aggregation (i.e., TGM2) of Tau. This makes them prime candidates for further investigation into their functions and use as therapeutic targets. Although much more research is required, the targeting of specific binding motifs involved in CaM regulation of these key enzymes is presented as a novel and precise therapeutic approach for the prevention of Tau phosphorylation and aggregation in various tauopathies.

## Data Availability

The original contributions presented in this study are included in the article. Further inquiries can be directed to the corresponding author.
